# Excessive intake of sugar: An accomplice of inflammation

**DOI:** 10.3389/fimmu.2022.988481

**Published:** 2022-08-31

**Authors:** Xiao Ma, Fang Nan, Hantian Liang, Panyin Shu, Xinzou Fan, Xiaoshuang Song, Yanfeng Hou, Dunfang Zhang

**Affiliations:** ^1^ Department of Biotherapy, State Key Laboratory of Biotherapy and Cancer Center, West China Hospital, Sichuan University, Chengdu, China; ^2^ Department of Rheumatology and Autoimmunology, The First Affiliated Hospital of Shandong First Medical University & Shandong Provincial Qianfoshan Hospital, Shandong Key Laboratory of Rheumatic Disease and Translational medicine, Shandong medicine and Health Key Laboratory of Rheumatism, Jinan, China

**Keywords:** macrophages, autoimmune disorders, Th17 cells (Th17), low-grade chronic inflammation, TGF-beta, IL-1beta

## Abstract

High sugar intake has long been recognized as a potential environmental risk factor for increased incidence of many non-communicable diseases, including obesity, cardiovascular disease, metabolic syndrome, and type 2 diabetes (T2D). Dietary sugars are mainly hexoses, including glucose, fructose, sucrose and High Fructose Corn Syrup (HFCS). These sugars are primarily absorbed in the gut as fructose and glucose. The consumption of high sugar beverages and processed foods has increased significantly over the past 30 years. Here, we summarize the effects of consuming high levels of dietary hexose on rheumatoid arthritis (RA), multiple sclerosis (MS), psoriasis, inflammatory bowel disease (IBD) and low-grade chronic inflammation. Based on these reported findings, we emphasize that dietary sugars and mixed processed foods may be a key factor leading to the occurrence and aggravation of inflammation. We concluded that by revealing the roles that excessive intake of hexose has on the regulation of human inflammatory diseases are fundamental questions that need to be solved urgently. Moreover, close attention should also be paid to the combination of high glucose-mediated immune imbalance and tumor development, and strive to make substantial contributions to reverse tumor immune escape.

## Introduction

It is well known that high-sugar consumption is a hallmark of the Western diet (1). Dietary sugars mainly refer to fructose and glucose which are naturally present in fruits and some vegetables (2, 3). Their molecular formula is C_6_H_12_O_6_ and they are isomers of each other (4). Fructose and glucose are both considered to be sweet sugars, yet fructose is the sweeter of the two. HFCS is a common sweetener and preservative made from the simple sugars’ fructose and glucose. HCFS-55 and HCFS-42, the most commonly utilized form that is used in beverages and baked goods, contains 55% and 42% fructose, respectively, with the remainder of the of the syrup being glucose (5). Since the 1970s, the amount of HFCS has increased in foods that are common within the Western diet (5, 6). The United States currently is the major user of HFCS, but HFCS is now produced throughout the world with factories on every continent except for Antarctica (5, 7). The consumption of these sugars, particularly in sugary soft beverages (SSB), became a major contributor to sugar intake, and the relationship between SSB and cardiometabolic diseases reflects the potential effects of fructose and glucose (8, 9).At the beginning of the twenty-first century, the U.S. Department of Agriculture reported that the consumption of soft drinks per capita in the United States had increased by about 500% over the past 50 years (10). To make matters worse, approximately 12% of infants consumed sugary sugar-sweetened beverages, a population who had a higher consumption of confectionaries and lower intake of fruits and vegetables only a couple of years later (10). In Brazil, consumption of sugary soft drinks roughly quadrupled from 1974 to 2003, and in 2009, Brazilian adults consumed about 100 ml/day of SSB (11, 12). In addition, in Europe, sugar consumption in different countries is between 7% and 25% of total energy intake (12). With the deepening of research on the relationship between high sugar diets and human health, the potential threat of high sugar diets to the incidence of noncommunicable diseases has become increasingly recognized (13).

A growing body of research suggests that excessive consumption of processed foods containing dietary sugars or HFCS is strongly linked to the development of obesity (14, 15), T2D (16, 17), metabolic syndrome (16) and cardiovascular disease (18). In 2004, Bray and his colleagues published a review article in the American Journal of Clinical Nutrition that drew attention to the potential relationship between sugar and obesity (19). This paper analyzed food consumption patterns using USDA food consumption tables from 1967 to 2000, and found that consumption of HFCS significantly outperformed changes in intake of any other food over that time period, ultimately confirming that HFCS consumption in high-calorie sweet drinks played a role in the obesity epidemic (20). As the study of sugar and obesity continues to deepen, researchers are looking at whether simple sugars, such as glucose and fructose, contribute to obesity. Some comprehensive information suggests that while both fructose and glucose contribute to weight gain (21), fructose intake is more likely to promote lipid deposition in visceral adipose tissue (VAT), while glucose consumption appears to favor subcutaneous adipose tissue (SAT) deposition (3). Other studies have shown that fructose intake appears to increase triglyceride concentrations in healthy male and decrease glucose tolerance and insulin sensitivity in obese older adults, compared with an equal-calorie glucose diet (3, 22). However, both intracellular triglyceride levels and insulin metabolism are associated with diabetes. Increased fructose or glucose intake is known to indicate a higher risk of T2D in adults, but the pathogenesis of the two is different (17). Glucose mediates the development of T2D through its high glycemic index, leading to interruption of insulin secretion (17). Fructose on the other hand is associated with a variety of factors, including weight gain, influence on insulin sensitivity, and fatty acid synthesis (23, 24). In addition, SSB made with HFCS can increase the risk of T2D by affecting blood sugar metabolism (25).

Another meta-analysis, which collected prospective cohort studies of 1 year or longer using a first-order linear mixed effects model, found a negative linear door-response relationship between SSBs and metabolic syndrome (RR 1.14 at 355 mL/d), confirming the association between sugary foods and the onset of metabolic syndrome (26). Relevant randomized controlled trials have also confirmed that people who drink at least one soft drink a day have a 44% higher risk of developing metabolic syndrome than people who do not drink soft drinks (27). Similarly, higher sugar intake is associated with cardiovascular disease. Analysis of The National Health And Nutrition Examination Survey (NHANES) III-related mortality cohort data shows that the intake of added sugar and SSB can lead to the occurrence of hypertension, stroke, coronary heart disease, and dyslipidemia, thereby increasing the risk of death (9, 18). In 1987, Hwang et al. studied Sprague-Dawley rats with fructose in their diet and found for the first time that a high-fructose diet was associated with hypertension (28). Subsequent studies confirmed that the increase in blood pressure caused by a high-fructose diet was due to the activation of the sympathetic nervous system (29, 30). Several other statistical studies conducted follow-up surveys of different populations and concluded that SSB intake is positively correlated with coronary heart disease (31–34), vascular events (35), heart failure (36), and stroke (37), but has nothing to do with subclinical atherosclerosis (38). Therefore, it is necessary to strengthen the social supervision of sugary processed foods. The World Health Organization (WHO) believes that sugars consumption varies with age and country, and their guidelines strongly recommend reducing sugar intake to less than 10% of total energy intake (10). The Scientific Advisory Committee on Nutrition in England (SACN) has also issued a similar policy, recommending that upper limit of sugar intake should not to exceed 5% of total energy intake (19). Despite this, it is difficult for many people of all ages to reduce,let alone eliminate, their intake of sugary drinks (10).

**Figure 1:**
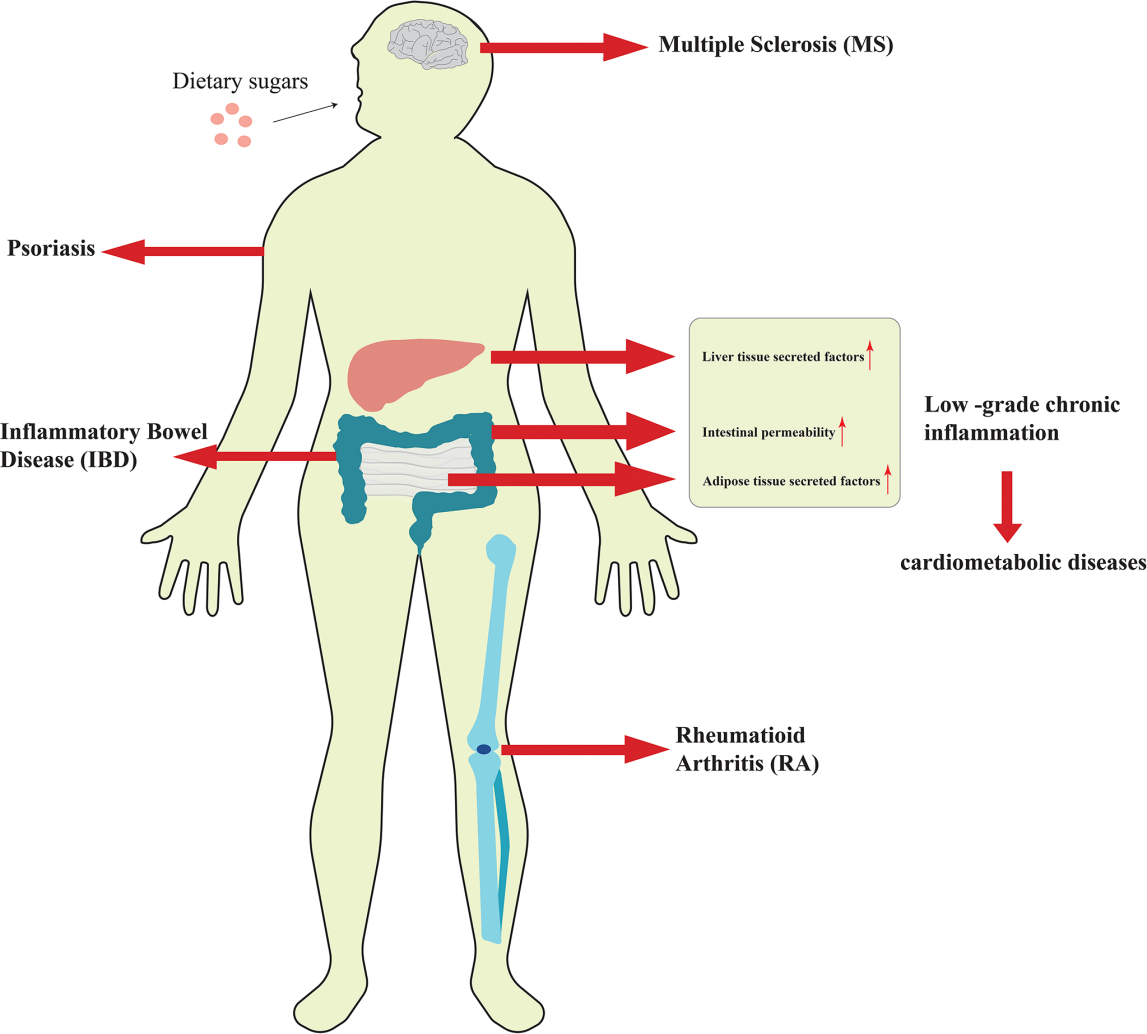
Excessive consumption of dietary sugars is closely related to the occurrence and development of inflammation.

Although the research on the relationship between dietary sugars and the above diseases has been relatively thorough, the impact of these sugars on inflammation was previously unknown. In recent years, as more researchers have explored the relationship between high-sugar diet and inflammation, people have found that excessive sugar intake is closely associated with the development of low-grade chronic inflammation and autoimmune diseases (**Figure 1**). Low-grade chronic inflammation has long been linked to obesity and increased body fat, and excess dietary sugar intake is a key contributor to obesity and weight gain. Autoimmune disease is a common disorder caused by the immune system attacking its own normal tissues. Although dietary structure is considered to be a key cause of autoimmune diseases, the impact and mechanism of dietary sugars on it has not been revealed until recently. Based on this, this paper reviews the effects and related regulatory mechanisms of excessive consumption of dietary sugars on inflammatory diseases discovered in recent years. By summarizing the current research progress, it has been revealed that dietary sugar is a key factor in inducing low-grade chronic inflammation, autoimmune diseases, and even neuroinflammation.

## Effects of dietary sugars on low-grade chronic inflammation

It has been shown that excessive intake of dietary sugars can cause metabolic disorders and induce the increase of inflammatory mediators and certain pro-inflammatory cytokines in various tissues, which leads to insulin resistance and low-grade chronic inflammation (39, 40). Low-grade chronic inflammation could be caused by factors secreted by adipose tissue, inflammatory factors secreted by liver tissue, and increased intestinal permeability, which may eventually lead to the development of cardiometabolic diseases (39, 41). Therefore, the association between high sugar intake and increased risk of chronic disease may be mediated in part by low-grade chronic inflammation. In low-grade chronic inflammation, the pro-inflammatory molecules mainly included Toll-like receptor 4(TLR-4), plasma C-reactive protein (CRP), interleukin-6 (IL-6), tumor necrosis factor-α (TNF-α), and monocyte chemotactic protein 1 (McP-1), E-selectin (E-selectin), plasminogen activator inhibitor 1 (PAI-1) as well as others (40, 42, 43). Several randomized trials have investigated the relationship between dietary sugars and systemic inflammation. Faizan et al. distributed beverages containing 50 grams of fructose, glucose, and sucrose to healthy subjects and found that all three increased blood lipid and hs-CRP levels, but fructose and sucrose were significantly more effective than glucose (44). A follow-up prospective trial of six 3-week dietary interventions in 29 healthy young men showed that low to moderate intake of SSBs containing HFCS had potentially harmful effects on low density lipoprotein (LDL) particles, fasting glucose, and hs-CRP (45). However, Jessica and colleagues found that there was no significant change in hS-CRP and IL-6 levels, markers of low-grade chronic inflammation, at the end of the diet period in normal-weight and obese adults who consumed four servings of beverages containing fructose, glucose or HCFS in addition to a standard diet over three eight-day periods. It was concluded that excessive consumption of fructose, HFCS, and glucose from SSBs over 8 days had no difference in low-grade chronic systemic inflammation in normal-weight and obese adults (39). Nor and his team came to similar conclusions. They found no significant differences in inflammatory biomarkers such as CRP, IL-1β, IL-6, and TNF-α in all dietary groups after 12 weeks in parallel trials of several high-fructose beverages (46). This contradiction may be caused by the age and physical condition of the subjects and the difference in sugar intake. In addition, other studies showed that lipocalin-2, e-selectin, McP-1 and PAI-1, all markers of systemic inflammation, were also up-regulated in high-fructose fed rats (42, 47).

Adipose tissue is one of the largest endocrine organs in the body and affects local and systemic immune function and metabolism by secreting inflammatory factors (43). Glucocorticoids are the key to the pathogenesis of monosaccharide-induced metabolic syndrome (48). In rats fed a high fructose diet, adipose tissue expressed more corticosterone (CORT), which was then offset by increased levels of macrophage migration inhibitor (MIF) (43, 48). The activity of nuclear factor -κB (NF-κB) decreased in adipose tissue, and the expression of inflammatory factor TNF-α did not change. In liver tissue, the level of 11β HSD1 protein was elevated, but did not affect intracellular CORT levels or downstream glucocorticoid signaling. Therefore, the activation of NF-κB was enhanced, and the level of pro-inflammatory factor TNF-α was increased (41). This could be interpreted as a tissue-specific result of the regulation of metabolic inflammation by high fructose intake. In another study in rats, fructose reduced fatty acid oxidation by decreasing liver peroxisome-proliferator-activated receptor α (PPAR-α) activity, ultimately leading to increased NF-κB activity (49). Fructose consumption, on the other hand, can induce liver and systemic inflammation through intestinal changes. It was found that fructose can promote the translocation of microbial substances from the intestinal tract to the portal vein circulation, activate the NF-κB and JAK2/STAT3 pathways through TLR4, and release inflammatory factors such as IL-1β, IL-6, and TNF-α (50, 51). At the same time, fructose intake can also increase intestinal permeability and promote the release of inflammatory factors to the liver, thereby increasing liver and systemic inflammation (52). The researchers also found that fructokinase, a key enzyme in fructose metabolism, plays an important role in inflammation caused by non-alcoholic fatty liver disease. Fructokinase knockout mice fed a high-sugar or high-fat diet were protected from liver inflammation and fibrosis, and the expression of inflammatory factors CD68, TNF-α, McP-1, smooth muscle actin, type I collagen, and TIMP1 was reduced (53). Similarly, liver inflammation and fibrosis also occurred in mouse models with low density lipoprotein (LDL) receptor defects that were fed the Western diet and liquid fructose (54). Another study showed that high fructose consumption can also have damaging effects on the hippocampus, an area of the brain important for learning and memory (55). The role of high fructose in hippocampal inflammation was confirmed by analysis of inhibition of phosphorylation of Ser 307 by hippocampal insulin receptor substrate 1 (IRS-1), protein levels of (NF-κB), and mRNA levels of related inflammatory factors (56).

## Effects of dietary sugars on autoimmune diseases

Autoimmune diseases (AID) are T cell-mediated inflammatory pathologies (57). Normally, the body’s immune system does not respond to its own components, known as autoimmune tolerance. AID is an immune pathological state in which the body’s autoimmune tolerance mechanism is deregulated or destroyed, resulting in damage or dysfunction of its own tissues and organs (13). The incidence of AID has increased in recent decades, but the reasons for this remain unclear. Current research shows that individual genetic susceptibility and environmental factors are closely related to the disease (58, 59). Although dietary changes, such as high salt intake (60, 61), are thought to be closely associated with increased incidence of AID, the effects and mechanisms of high-sugar diets include rheumatoid arthritis (RA), multiple sclerosis (MS), psoriasis, and inflammatory bowel disease (IBD) have only been uncovered in recent years (13, 57, 62).

### Effects of dietary sugars on rheumatoid arthritis

Rheumatoid arthritis (RA) is one of the most common systemic, chronic, autoimmune diseases caused by genetic, environmental, and endogenous factors (63). It is characterized by systemic inflammation and persistent synovitis (64). In recent years, numerous studies have shown that sugar-sweetened beverages play a key role in the pathogenesis of RA (63, 65, 66). In a follow-up survey, researchers found that women who drank $1 a day of sugar-sweetened beverages had an increased risk of seropositive RA compared with women who didn’t drink sugar-sweetened beverages, with a greater risk among women over 55 (64). A subsequent study showed that the reason why sugar-sweetened beverages can cause RA, in addition to their important role in the autoimmune mosaic, is that it is more likely to alter the microbiome, thereby affecting downstream inflammatory pathways (63). High consumption of glucose, fructose, and sugar-sweetened beverages is known to reduce the beneficial flora in the gut, especially Prevotella, which has been found to be associated with the pathogenesis of RA (67). In addition, the Mediterranean diet has been shown to reduce the incidence of diseases such as RA compared to a high-sugar Western diet (66, 68).

### Effects of dietary sugars on multiple sclerosis

Multiple sclerosis (MS) is an autoimmune disease of the central nervous system with symptoms that affect multiple systems throughout the body, including visual impairment, movement disorders, fatigue, cognitive and emotional disturbances, pain, and more (69). In MS, immune cells cross the blood-brain barrier (BBB)into the central nervous system to attack self-antigens, resulting in BBB disruption and loss of oligodendrocytes and myelin, leading to axonal degeneration and permanent neurological deficits (69, 70). Many studies have shown that lifestyle choices, including diet, can affect some of the symptoms of MS, and it seems that people with MS can relieve their symptoms by improving their eating habits (70). For example, one study noted that subjects with multiple sclerosis ate more carbohydrates than the control group, but there was no difference in BMI between the two groups. The researchers attributed this to the small sample size used in the study (71). Although the effect of a high-sugar diet on MS has not been confirmed in clinical studies, it has been found that high-glucose and high-sucrose diets can aggravate the disease progression of experimental autoimmune encephalomyelitis (EAE) in a disease model of MS (i.e., the EAE model) (13, 57). Both studies found that high sugar intake increased the proportion of CD4^+^ cells in EAE mice and exacerbated neuroinflammation in the brain and spinal cord, but both studies looked at the deleterious effects of high sugar diets from different pathogenic mechanisms and confirmed two things. On the one hand, high-glucose diet can directly act on CD4^+^ T cells, by inducing T cells to differentiate into Th17 cells, thereby increasing the proportion of Th17 cells in EAE mice (13). On the other hand, a high-sugar diet stimulated Th17 cell differentiation and exacerbated EAE by altering the colony structure of the gut microbiome (57).

### Effects of dietary sugars on Psoriasis

Psoriasis is a chronic inflammatory skin disease characterized by abnormal proliferation and differentiation of epidermal keratinocytes (72, 73). Previous studies have shown that inflammatory adipocytokines such as IL-6 and TNF-α formed in visceral adipose tissue are key cytokines in the pathogenesis of psoriasis, so it is believed that psoriasis is related to obesity (57, 74, 75). However, new research data suggests that dietary components (simple sugars and fats), rather than obesity itself, exacerbate psoriasis (76). The researchers found that the western diet activated the interleukin 23(IL-23) signaling pathway compared with the normal diet before the mice gained weight, further increasing the production of IL-17A in γδT cells after IL-23 stimulation (76). The cytokine IL-17A is necessary for the comprehensive development of skin inflammation (77). Meanwhile, IL-23 overexpression resulted in decreased microbial diversity and pronounced dysbiosis in mice fed the Western diet (78). Even more surprising, when the mice were switched from a western diet to a standard one after IL-23 was released, skin inflammation was reduced and the gut microbiota partially reversed (78). Therefore, based on the available data, we believe that the dysbiosis of the gut microbiota induced by short-term Western dietary intake contributes to the enhancement of psoriasis, and healthy eating pattern with less sugar should be considered for patients with psoriatic skin disease (79).

### Effects of dietary sugars on inflammatory bowel disease

Inflammatory bowel disease (IBD) is a chronic inflammatory gastrointestinal disease that mainly includes two subtypes, Crohn’s disease and ulcerative colitis (80). It occurs due to the interaction of multiple factors such as genetics, microbes, immune factors, modern lifestyle, and diet (81, 82). Existing research suggests that IBD affects disease severity by affecting changes in the microbial composition of the gut microbiota, while colitis microbiota shifts and alters colitis susceptibility in recipients (83). The commensal gut flora and mucus layer in the gut are known to be critical for homeostasis, as it prevents the invasion and adhesion of pathogenic microorganisms and helps maintain the integrity of the gut barrier (84). According to statistics, the incidence of IBD in Western countries is increasing, especially among children in the same period (85), indicating that the occurrence of IBD is related to Western diet and lifestyle. In recent years, IBD has also become a global health problem due to the simultaneous rise of Western diets (ie, diets high in fat and refined sugar) around the world. Recent clinical and experimental studies suggest that a high-fat diet may be a trigger for IBD, but the role of high sugar in the pathogenesis of IBD remains controversial. A landmark study shows that type 2 diabetes can lead to intestinal barrier dysfunction through transcriptional reprogramming of intestinal epithelial cells and altered tight adhesion junction integrity; it can also increase disease by causing changes in gut microbial metabolism susceptibility (86). Additionally, population-based studies have shown that about 10% of people with IBD believe that eating sugary foods trigger flare-ups and make their symptoms worse (87). In some prospective studies, consumption of HFCS and SSB have also been found to be positively associated with the risk of IBD (88–90). Taken together, the researchers believe that sugar is closely related to the composition of the gut microbiome and the occurrence and development of IBD.

## Mechanisms by which dietary sugars affects inflammation

**Figure 2:**
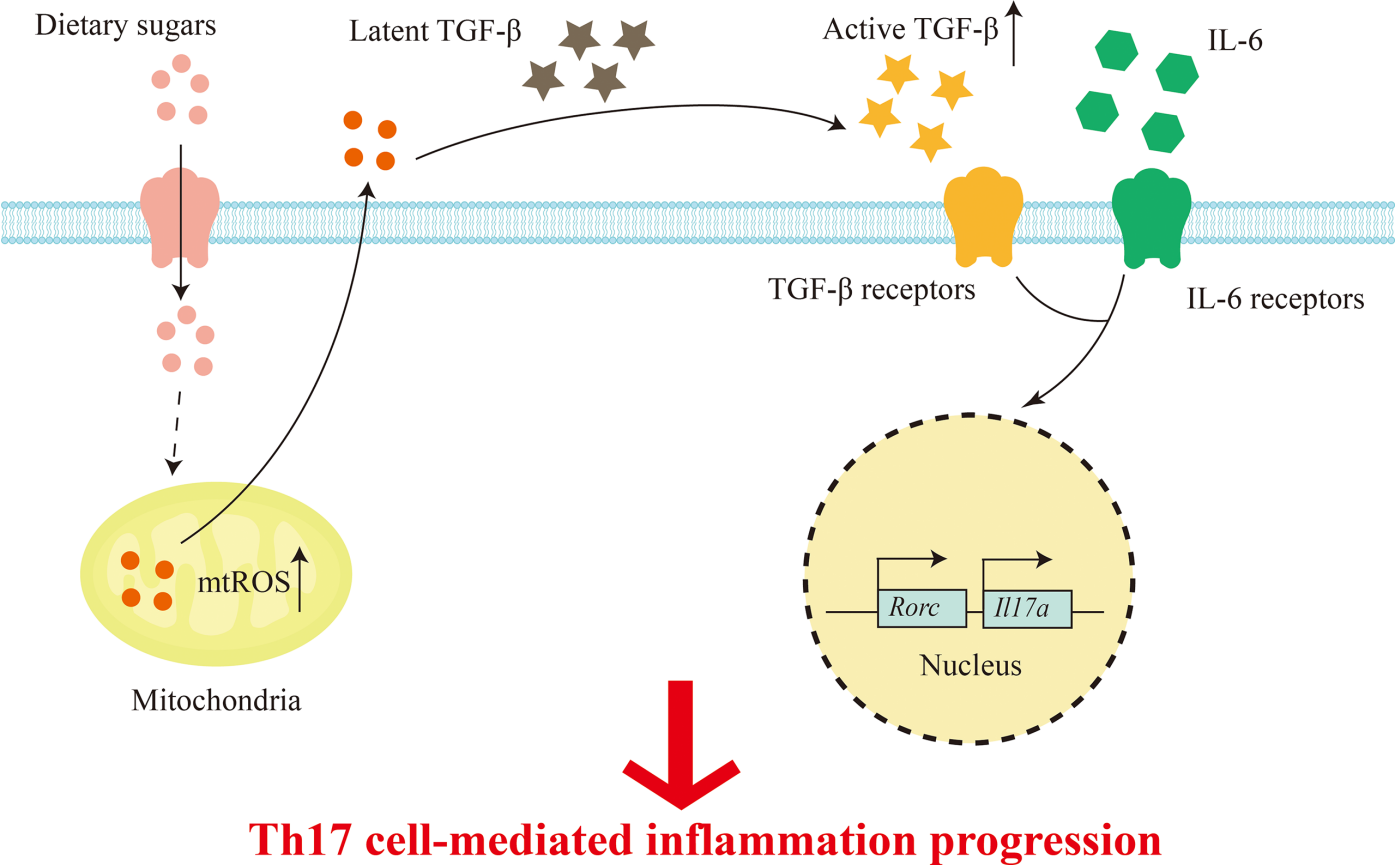
Dietary sugars-mediated T cell inflammation. Excess dietary sugars may activate TGFβ through mtROS post entering T cells, and together with IL-6 in the immune microenvironment, the expression of transcription factor RORγt is induced to promote Th17 cell differentiation.

The high glucose environment is inextricably linked with the immune system, which plays an important role in immune signal and immune cell function (91).Previous study has found that high levels of glucose may lead to impaired immune system function and pathological conditions. Innate immune macrophages, dendritic cells, and specific immune cells T cells and B cells migrate to the site of infection to protect the immune system (92). T cells are the key to cell-mediated immunity. Conventional T cells, also called αβT cells, can be differentiated into effector CD8^+^ cytotoxic subsets and CD4^+^ helper T cell subsets, including Th1, Th2, Th17, Tr1, Tfh, Th9, and immunosuppressive Treg cells, respectively, under the stimulation of antigen (91, 93, 94). Thaís and his colleagues used lymphocyte culture and analysis of its CD4^+^ and CD8^+^ subpopulations to confirm that high concentrations of fructose can reduce lymphocyte subcomponents, resulting in a decrease in the total number of lymphocytes (95). In addition, hypertonic glucose in the peritoneal dialysis (PD) range has been reported to induce interleukin-17 (IL-17) polarization in a mitochondrial reactive oxygen species (mtROS)-dependent manner (96). Subsequently, Zhang et al. demonstrated that high glucose can activate TGF-β through ROS, and subsequently promote Th17 cell differentiation with the participation of IL-6, thereby aggravating autoimmune disease, in T cell metastasis and experimental autoimmune encephalomyelitis (EAE) induced colitis mouse models (13, 62). Therefore, high amounts of dietary sugars can lead to T cell-mediated inflammation (**Figure 2**). Recent studies have found that dietary components also have regulatory effects on B cells, but it is not clear which nutrients affect B cells. In order to solve this problem, Tan and his colleagues used statistical modeling to study the effects of carbohydrates, fats, and proteins on B cells, and found that carbohydrates have a great regulatory effect on B cell proliferation (97). In addition, they showed that it is glucose, but not fructose, that supports B lymphocyte generation and development, while protecting B lymphocytes from early apoptosis through activation of the mammalian target of rapamycin signaling pathway (mTOR) (97). Additionally, a recent study mentioned the effects of a high-fructose diet (HF), high-fat diet (HFD), or both (HFHF) on leptin and ROS, but it appears that only HFHF-fed mice developed hyperglycemic symptoms, oxidative stress, and steatosis (inflammation and fibrosis), whereas HF caused only transient increases in leptin and C-peptide (98).

**Figure 3:**
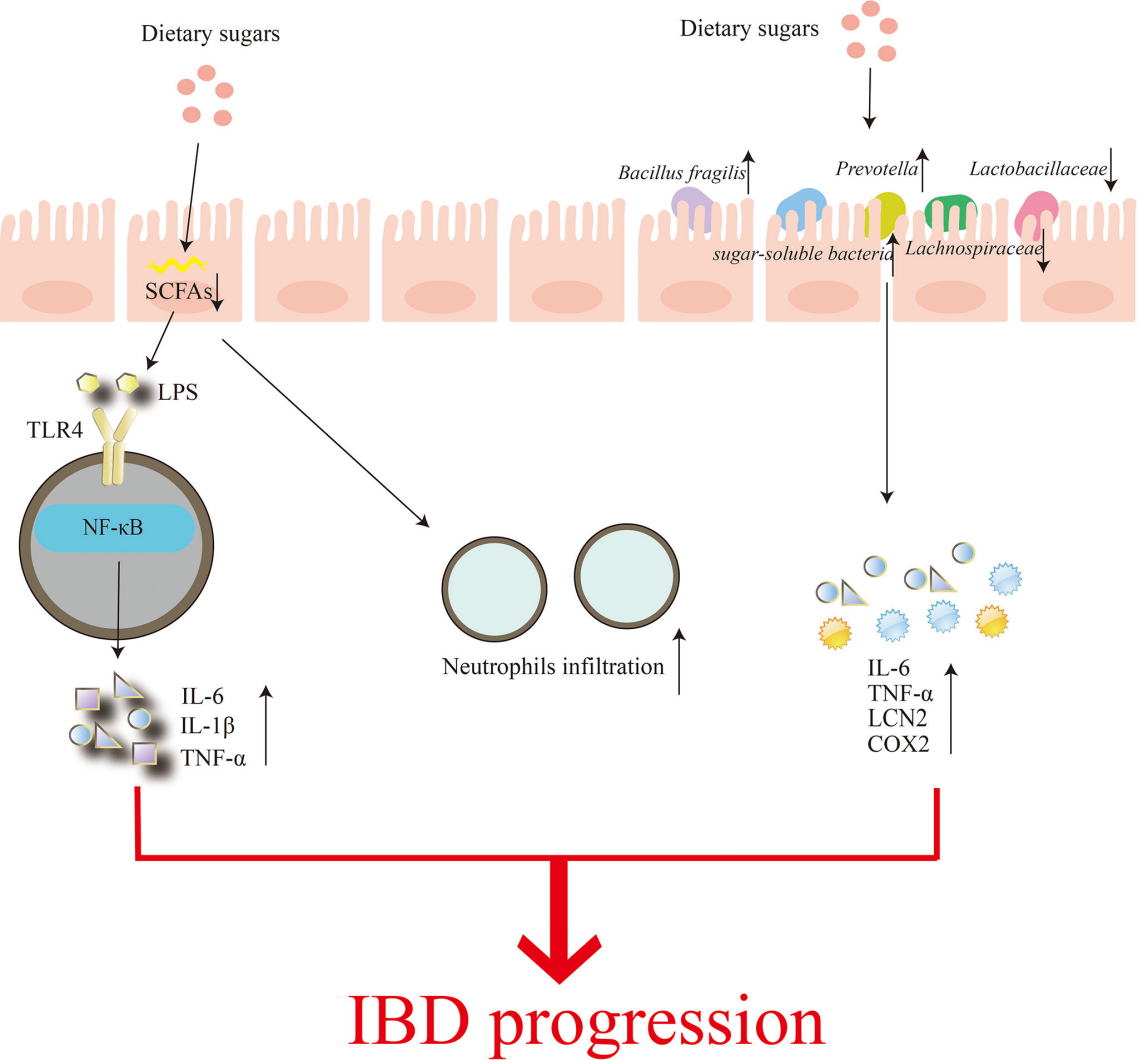
Regulation of the gut microbiome by dietary sugars. Excessive consumption of dietary sugars reduces the production of short-chain fatty acids in the gut, which can lead to impaired gut barriers. This results in a rapid increase in infiltration of neutrophils while accelerating the transfer of Parabacteroides, ie, lipopolysaccharide (LPS). The binding of LPS to TOLL-like receptor 4 (TLR4) activates the nuclear factor-κB (NF-κB) signaling pathway, and finally induces the production of inflammatory factors IL-6, IL-1β and TNF-α. On the other hand, the excessive dietary sugar content makes Bacillus fragilis and Prevotella abundant, thereby destroying the intestinal mucosa. In the meanwhile, the relative abundance of sugar-soluble bacteria Sutterellaceae increased while the abundance of Lachnospiraceae and Lactobacillaceae, which belonged to Firmicutes, decreased, eventually increasing the levels of inflammatory cytokines IL-6, TNF-a, Lcn2 and Cox2. Increased neutrophil infiltration and inflammatory factor production aggravate the occurrence and development of IBD.

The gut microbiome has also been the focus of research into the effects of dietary sugars on inflammation. It mainly includes two aspects: (1) High consumption of sugars reduces microbial diversity and leads to depletion of luminal short-chain fatty acids (SCFAs) (99). SCFAs can affect the recruitment of colonic regulatory T cells and the antibacterial activity of macrophages, thereby affecting the intestinal mucosal immune system (100). The damaged intestinal barrier is unable to prevent the invasion of pathogenic microorganisms, enabling the transport of E. coli-derived (LPS), etc., which are recognized by their specific receptors, such as TOLL-like receptor 4 (TLR4), and activate downstream NF-κB signaling pathway induces increased levels of inflammatory factors IL-6, IL-1β and TNF-α and more neutrophil infiltration, leading to more severe colitis (99). (2) Shahanshah and colleagues suggest that high glucose can increase the levels of inflammatory cytokines IL-6, TNF-a, Lcn2 (Lcn2), and Cox2 (Ptgs2) by altering gut microbiota composition, mucosal association, and functional activity, thereby aggravating the progression of inflammatory bowel disease (1). In this study, Shahanshah found that mucolytic bacteria, such as Bacillus fragilis and Prevotella, were abundant in mice fed a high-glucose diet, while the relative abundance of the sugar-soluble bacteria Sutterellaceae, capable of transplanting to the epithelial barrier and inducing an inflammatory response was increased. In contrast, the abundance of Lachnospiraceae and Lactobacillaceae belonging to Firmicutes decreased. Lachnospiraceae have been shown to suppress inflammation, while Lactobacillaceae are able to maintain intestinal homeostasis by inducing anti-inflammatory cytokines and protecting the intestinal epithelium from pathogens. Similarly, dietary fructose can induce intestinal inflammation by increasing intestinal cell permeability and promoting the growth of intestinal bacteria (101)(**Figure 3**). In addition, the effect of high fructose on the severity of IBD was abolished when gut bacteria were substantially reduced, suggesting that the changes in gut microbial composition and IBD effects of high glucose are transferable (102).

**Figure 4:**
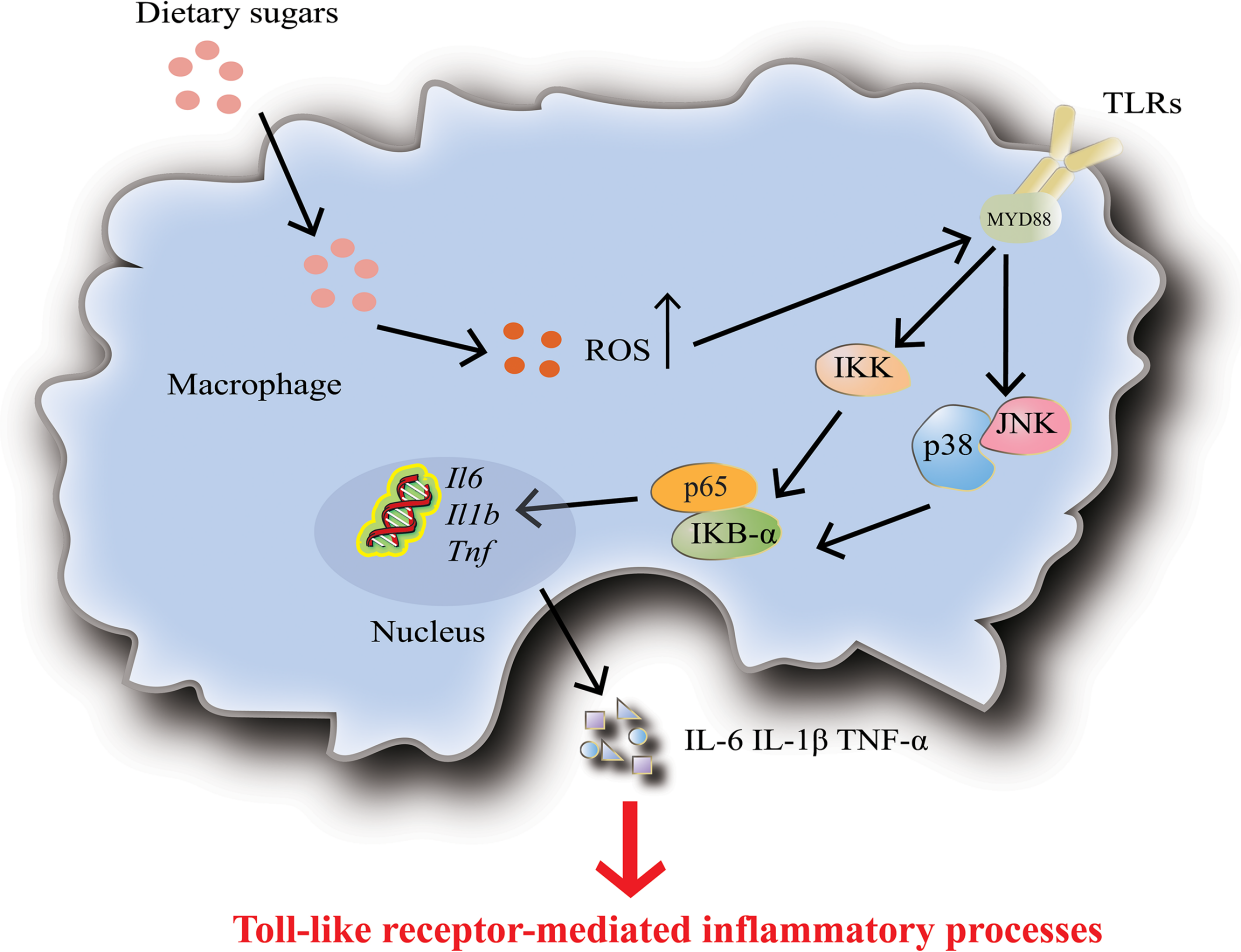
Dietary sugars-mediated inflammation in macrophages. High levels of dietary sugars lead to increased TOLL-like receptor 4 (TLR4) activity, which subsequently activates downstream the nuclear factor-κB (NF-κB) and MAPK signaling pathways, thereby promoting the upregulation of inflammatory factors IL-6, IL-1β and TNF-α. In addition, dietary sugars-mediated inflammation in dendritic cells and neutrophils is also accomplished by activating TLR4.

Macrophages are one of the most specialized antigens presenting cells, whose main functions are to secrete cytokines, phagocytose, and present antigens to T cells (92). The researchers found that high glucose levels induce increased expression and activity of Toll-like receptors (TLRs), which then activate NF-κB and MAPK signaling pathways through ROS/RNS and superoxide production, leading to macrophage activation and release of inflammatory factors (**Figure 4**) (103, 104). High doses of glucose can induce superoxide anion production in macrophages or monocytes and promote the release of monocyte inflammatory cytokines, which up-regulate innate immune system receptors (such as TLRs) by activating NF-κB (105). Complementary to this, high glucose conditions impair neutrophil mobilization which is due to elevated TLRs expression (106).

## Discussion

The leading cause of death in patients with diabetes is related to its accompanying complications, such as diabetic retinopathy, obesity, and cardiovascular disease (107). Inflammation and immune abnormalities are triggers for T1D and T2D and its associated complications (108, 109). When the body is attacked by an antigen, innate immune macrophages and specific immune lymphocytes are triggered to migrate to the site of infection to function, however, large amounts of glucose may lead to impaired immune system function (110). Therefore, high glucose induces a series of complications by suppressing the effective adaptive immune response generated by macrophages and T cells.

Studies have shown that dietary monosaccharide consumption is associated with T2D and cardiovascular disease, and obesity increases the risk of these diseases (110). Meanwhile, low-grade chronic inflammation is also strongly associated with obesity (39). Therefore, the association between dietary sugars and increased risk of chronic disease may be mediated by low-grade chronic inflammation. Another randomized controlled trial showed no difference in the effects of fructose, glucose, or HFCS on obesity and systemic or adipose tissue inflammation in normal-weight adults (39). With the increasing consumption of these dietary sugars and their beverage mixes, more people around the world are suffering from systemic inflammation. A large number of studies have shown that natural small molecules widely present in plants have an inhibitory effect on systemic inflammation caused by excessive intake of dietary sugars. Studies have shown that curcumin inhibits inflammation caused by high fructose through multiple pathways. In male Wistar rat inflammatory model, curcumin can inhibit the elevation of malondialdehyde (MDA) and total oxidation state (TOS) in skeletal muscle and the expression of extracellular kinase 1/2 (ERK1/2) and P38 proteins of MAPK family members (111). Curcumin and allopurinol inhibit liver inflammation by upregulating the Mir-200A-mediated TXNIP/NLRP3 inflammasome pathway (112). Epatechin (113), astaxanthin (114), morin (115), and juglanin (116) reduce systemic inflammation by inhibiting the release of inflammatory factors IL-6, IL-1β, and TNF-α through downstream cascades activated by TLR4 such as NF-κB, MAPK, or JAK2/STAT3, and betulinic acid ameliorates inflammation and oxidative stress induced by high fructose diet through PIK and Akt pathways (117). In addition, S-methylcysteine (SMC) (118), spinach nitrate (119) and red ginseng mulberry leaf (MPM) (120) can inhibit inflammation induced by dietary monosaccharide overdose by inhibiting the expression of low grade chronic inflammatory markers such as serum C-reactive protein, tumor necrosis factor A, and interleukin-6 e-selectin.

Autoimmune disease is an abnormal immune response in which the immune system attacks the body’s normal tissues, resulting in the chronic destruction of these tissues and severely reducing the patient’s quality of life (13). Consumption of glucose-rich foods and beverages is very common in the West and may also be a key cause of the breakdown of metabolic and immune self-tolerance (62). In the new mouse model, autoimmune disease in mice can be largely alleviated if a Western diet is switched to a normal diet (121). Therefore, a reasonable and balanced dietary recommendation (low fat, low sugar) is essential for patients with autoimmune diseases. A Mediterranean diet has been proven to be more conducive to the recovery of patients with autoimmune diseases than the Western diet (63, 79). In addition to improving diet, it is hoped that dietary restrictions can improve the effects of autoimmune disease and inflammation. Recently, Dixit and his team found that insisting on a 14% reduction in long-term calorie intake can help restore thymus function, increase thymus volume, and improve the ability of the thymus to generate T cells, thereby improving immune function that typically declines with age. Phospholipase PLA2G7 may play an important role in this mechanism (122). More intriguingly, Bukhari and his colleagues found that mothers’ high-fructose diets influenced neonatal immunity and altered anxiety behavior and inflammation in adolescence and adulthood (111). The study suggests that maternal diet may alter peripheral inflammation in newborns, which in turn affects anxiety-like behavior and peripheral inflammation during adolescence. These findings reveal the lasting effects of a mother’s diet on her offspring’s immune system, meaning that the mother’s diet is crucial for their child. Recently, it was demonstrated that excessive consumption of HFCS is associated with colon cancer development (123). In the study, mice fed with HFCS had significantly increased tumor size. This means that excess dietary sugar might be closely related to the development of tumors. However, whether immune regulation plays any key role in the tumor microenvironment remains to be explored. Overall, most of the studies were performed with mouse models, limiting the clinical applicability of these findings. Therefore, it is urgent to reveal the roles of excessive intake of hexose in the regulation of human inflammatory diseases in the future.

## Author contributions

XM wrote the manuscript. FN, HL, PS, XF and XS edited the manuscript. YH and DZ supervised the work, and edited the manuscript. All authors contributed to the article and approved it for publication.

## Funding

This work was supported by the Key Project of the Science and Technology Department of Sichuan Province (NO. 2022YFH0100), the National Natural Science Foundation of China (NO. 82171829), the 1·3·5 Project for Disciplines of Excellence, West China Hospital, Sichuan University (NO. ZYYC21012), the Fundamental Research Funds for the Central Universities (20822041E4084), Special Fund for Flow Cytometry Lymphocyte Subgroups of Shandong Provincial Medical Association (YXH2022ZX03223), and Shandong Medical and Health Technology Development Funds (2014WS0361).

## Acknowledgments

DZ sincerely wants to commemorate Dr. Sang-A Park, who passed away suddenly on January 22, 2018.

## Conflict of interest

The authors declare that the research was conducted in the absence of any commercial or financial relationships that could be construed as a potential conflict of interest.

## Publisher’s note

All claims expressed in this article are solely those of the authors and do not necessarily represent those of their affiliated organizations, or those of the publisher, the editors and the reviewers. Any product that may be evaluated in this article, or claim that may be made by its manufacturer, is not guaranteed or endorsed by the publisher.
